# Involvement of Salicylic Acid on Antioxidant and Anticancer Properties, Anthocyanin Production and Chalcone Synthase Activity in Ginger (*Zingiber officinale* Roscoe) Varieties

**DOI:** 10.3390/ijms131114828

**Published:** 2012-11-13

**Authors:** Ali Ghasemzadeh, Hawa Z. E. Jaafar, Ehsan Karimi

**Affiliations:** Department of Crop Science, Faculty of Agriculture, University Putra Malaysia, Serdang 43400, Selangor, Malaysia; E-Mail: ehsan_b_karimi@yahoo.com

**Keywords:** flavonoids, salicylic acid, chalcone synthase, HPLC, FRAP, DPPH, anticancer, Halia Bara

## Abstract

The effect of foliar application of salicylic acid (SA) at different concentrations (10^−3^ M and 10^−5^ M) was investigated on the production of secondary metabolites (flavonoids), chalcone synthase (CHS) activity, antioxidant activity and anticancer activity (against breast cancer cell lines MCF-7 and MDA-MB-231) in two varieties of Malaysian ginger, namely Halia Bentong and Halia Bara. The results of high performance liquid chromatography (HPLC) analysis showed that application of SA induced the synthesis of anthocyanin and fisetin in both varieties. Anthocyanin and fisetin were not detected in the control plants. Accordingly, the concentrations of some flavonoids (rutin and apigenin) decreased significantly in plants treated with different concentrations of SA. The present study showed that SA enhanced the chalcone synthase (CHS) enzyme activity (involving flavonoid synthesis) and recorded the highest activity value of 5.77 nkat /mg protein in Halia Bara with the 10^−5^ M SA treatment. As the SA concentration was decreased from 10^−3^ M to 10^−5^ M, the free radical scavenging power (FRAP) increased about 23% in Halia Bentong and 10.6% in Halia Bara. At a concentration of 350 μg mL^−1^, the DPPH antioxidant activity recorded the highest value of 58.30%–72.90% with the 10^−5^ M SA treatment followed by the 10^−3^ M SA (52.14%–63.66%) treatment. The lowest value was recorded in the untreated control plants (42.5%–46.7%). These results indicate that SA can act not only as an inducer but also as an inhibitor of secondary metabolites. Meanwhile, the highest anticancer activity against MCF-7 and MDA-MB-231 cell lines was observed for H. Bara extracts treated with 10^−5^ M SA with values of 61.53 and 59.88%, respectively. The results suggest that the high anticancer activity in these varieties may be related to the high concentration of potent anticancer components including fisetin and anthocyanin. The results thus indicate that the synthesis of flavonoids in ginger can be increased by foliar application of SA in a controlled environment and that the anticancer activity in young ginger extracts could be improved.

## 1. Introduction

**Figure 1:**
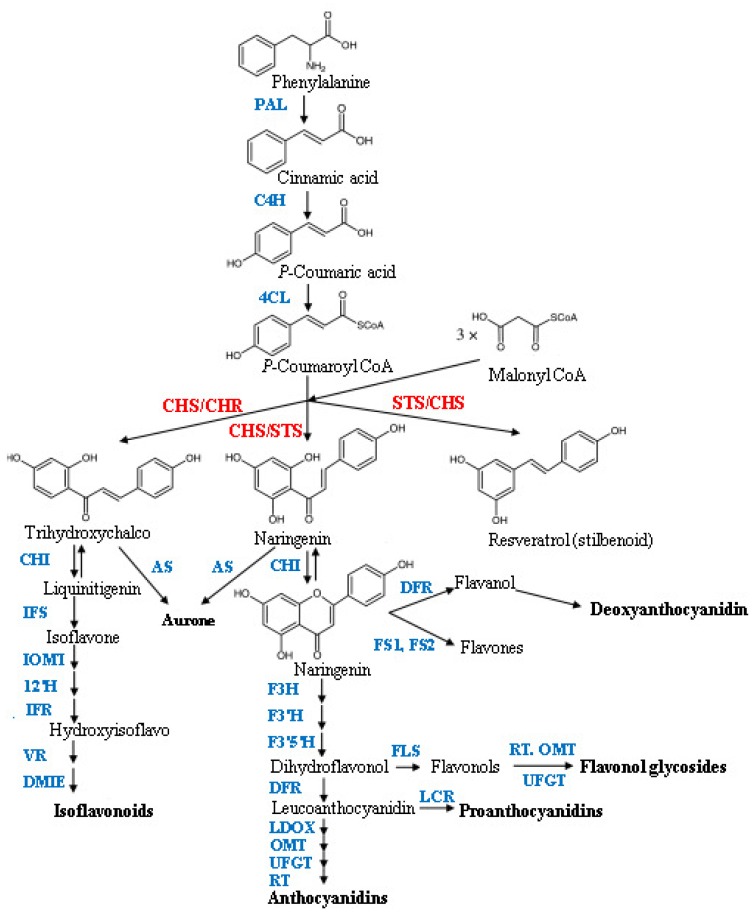
Flavonoid biosynthetic pathway. ANS, anthocyanidin synthase; AS, aureusidin synthase; C4H, cinnamate-4-hydroxylase; CHR, chalcone reductase; DFR, dihydroflavonol 4-reductase; DMID, 7,2′-dihydroxy,4′-methoxyisoflavanol dehydratase; F3H, flavanone 3-hydroxylase; F3′H, flavonoid 3′ hydroxylase; F3′5′H, flavonoid 3′5′ hydroxylase; FS1/FS2, flavone synthase; I2′H, isoflavone 2′-hydroxylase; IFR, isoflavone reductase; IFS, isoflavone synthase; IOMT, isoflavone *O*-methyltransferase; LCR, leucoanthocyanidin reductase; LDOX, leucoanthocyanidin dioxygenase OMTO-methyltransferase; PAL, phenylalanine ammonia-lyase; RT, rhamnosyl transferase; UFGT, UDP flavonoid glucosyl transferase; VR, vestitone reductase; STS, stilbene synthase; FLS, flavanol synthase [16].

Salicylic acid (SA) is a phenolic compound capable of enhancing plant growth and yield in some plants. SA acts as a potential non-enzymatic antioxidant, as well as a plant growth regulator, and plays an important role in regulating a number of plant physiological processes and bioactive compounds production [1]. Plants are potential sources of natural bioactive compounds which occur as secondary metabolites. They absorb sun light and produce high levels of oxygen and secondary metabolites during photosynthesis. Flavonoids are an important group of secondary metabolites and are a source of bioactive compounds in plants [2]. They are also a kind of natural product with antioxidant properties capable of scavenging free superoxide radicals, having anti-aging properties as well as reducing the risk of cancer. Sung-jin *et al.*[3] showed that some flavonoid components in green tea are effective in inhibiting cancer or induce mechanisms that may kill cancer cells and inhibit tumor invasion. It was found that flavonoids reduced blood-lipids and glucose, and enhanced human immunity [4]. Currently, flavonoids are extracted among other sources, from ginkgo leaves [5], kudzu roots [6], lotus leaves [7] and ginger rhizomes and leaves [8]. Chalcone synthase enzyme (CHS) is a key entry enzyme committed to the production of flavonoids in plants. Naringenin, the product of CHS activity is the initiator of a large variety of secondary metabolites including flavonoids, isoflavonoids, anthocyanins and phloroglucinols [9]. CHS condenses three malonyl-CoA molecules with cinnamoyl-CoA to produce chalcone. This condensation is the main pathway for the production of flavonoids (Figure 1), [10]. Ginger is one of the traditional folk medicines and it is widely used in cooking in Malaysia. It is extensively used overall especially in Asia and contains several interesting bioactive constituents which possess health promoting properties. Ginger is regarded as an important resource with a wide range of high flavonoid components, is low priced [11,12], and therefore serves as a cheap and important food material. Food composition and food additives play a major role in providing the required antioxidants for the body, although traditionally, spices such as ginger are commonly used in food preparations to improve flavor and taste. Previous studies by the current authors demonstrated potent antioxidant and anticancer activities of Malaysian ginger (Halia Bara and Halia Bentong) [13–15]. However, the impact of foliar application of SA on the production of biopharmaceuticals in Malaysian herbs has not been widely investigated. This needs to be understood, especially when the objective is production optimization with regard to herbal chemistry. Furthermore, information on SA effects on the chemistry of Malaysian ginger varieties is not available. Such data would be useful to provide information on the availability of high levels of beneficial components.

This study was therefore designed to examine the effects of foliar application of SA on the production of secondary metabolites in two varieties of Malaysian ginger (*Zingiber officinale*), namely H. Bentong and H. Bara and to evaluate CHS enzyme activity. In addition *in vitro* antioxidant and anticancer properties of the extracts against breast cancer cell lines were also investigated.

## 2. Results and Discussion

### 2.1. HPLC Analysis of Flavonoid Compounds

Results of high performance liquid chromatography (HPLC) analysis of flavonoids and phenolic acids are present in Table 1. Leaf extracts of Malaysian ginger, especially the variety H. Bara contained considerably (*p* ≤ 0.05) high amounts of rutin (0.893 mg g^−1^ DW) and apigenin (0.384 mg g^−1^ DW). SA application reduced rutin production in H. Bara (6.8%) and H. Bentong (21.8%). According to the data obtained, the concentration of some flavonoids (e.g., rutin, apigenin) decreased significantly in plants treated with different concentrations of SA (Table 1). High concentrations of these flavonoids were found in the control plants. Conversely, concentration of naringenin, fisetin and morin increased significantly in both varieties when treated with different concentrations of SA. The interesting finding was that application of SA in both varieties induced synthesis of fisetin and anthocyanin which were not detected in the control plants. Highest levels of anthocyanin (0.442 mg g^−1^ DW) and fisetin (0.359 mg g^−1^ DW) were observed in leaf extracts of H. Bara treated with 10^−5^ M of SA. Obinat *et al*. [17] also reported that production of anthocyanin was induced in cultured grape cells following foliar application of SA. Myricetin has antioxidant properties and *in vitro* research suggests that various concentrations of myricetin can modify LDL cholesterol and enable increased uptake by white blood cells [18]. In the present study production of myricetin was enhanced in ginger varieties treated with SA compared to control plants. High levels of this potent antioxidant compound were observed in H. Bara (0.112 mg g^−1^ DW) treated with 10^−5^ M SA. Morien is a rare yet well known flavonoid component of plants and acts as a chemo-preventive agent *in vitro* and *in vivo* against oral carcinogenesis [19,20]. The importance of morin and related compounds as anti-tumour drugs has also been widely recognized [21]. A high content of morin (0.193 mg g^−1^ DW) was obtained in extracts of H. Bara treated with 10^−5^ M SA. According to HPLC analysis, it could be concluded that the application of SA induced synthesis of some flavonoids, while conversely inhibiting production of some other flavonoids in ginger. Our results suggest the ability of SA application to modify or alter both the profile and the concentration of flavonoids in ginger.

### 2.2. Chalcone Synthase Enzyme (CHS) Activity

**Figure 2:**
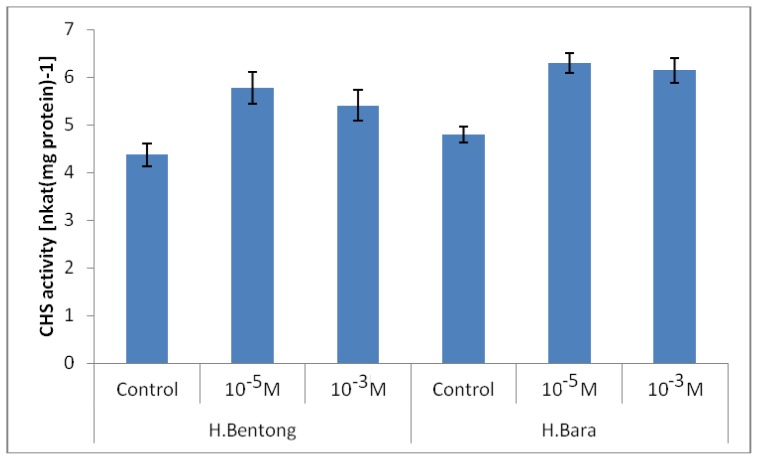
Chalcone synthase enzyme (CHS) activity in two ginger varieties treated with different concentration of salicylic acid (SA). Error bar represents standard error of means.

According to the results CHS activity was influenced by SA concentration (*p* ≤ 0.01; Figure 2). In both varieties treated with SA, CHS activity was found to be consistently higher in ginger treated with 10^−5^ M SA with values ranging between 5.77 and 6.30 nkat mg protein^−1^ than in gingers treated with 10^−3^ M SA, which recorded CHS activity of 5.40 to 6.14 nkat mg protein^−1^. Plants not treated with SA showed the lowest values for CHS activity which were registered between 4.37 and 4.80 nkat mg protein^−1^. The present study showed that with SA application the activity of CHS was enhanced. This is basically due to the fact that CHS is a precursor to flavonoids biosynthesis [22,23]. The increase in CHS activity is usually followed by an increase in C/N ratio due to the enhanced growth rate using SA. Results of recent studies suggest that increases in the C/N ratio in plants are an indication of increases in the synthesis of secondary plant metabolites, especially flavonoids [24,25].

It is hypothesized that the increase in production of anthocyanin and flavonoids in the present work could be attributed to an increase in CHS activity in SA treated plants. Our results are consistent with Compos *et al*. [10] who reported SA induced CHS activity in beans. Ozeki *et al.*[26] pointed out that change in CHS activity rather than PAL activity (also involving enzymes for flavonoid synthesis) was correlated with changes in anthocyanin accumulation under various culture conditions. CHS is the first enzyme to switch from phenylpropanoid metabolism to flavonoid metabolism and is believed to be a key enzyme in this system [27]. These findings together with evidence for channeling between SA concentration and CHS activity in the general phenylpropanoid pathway [16], indicate that the organization of these systems are important in the understanding of how plant metabolism is regulated.

### 2.3. Antioxidant Activity

#### 2.3.1. Ferric Reducing Antioxidant Potential (FRAP) Assay

Several methods have been used to measure the total antioxidant capacity of herbs, including FRAP assay, which has been adopted in this study. The FRAP assay depends on the reduction of ferric tripyridyltriazine (Fe (III)-TPTZ) complex to the ferrous tripyridyltriazine (Fe (II)-TPTZ) by a reductant at low pH. The reducing power of the leaf extract of young ginger was in the range of 476.22–793.26 μm of Fe (II)/g dry weight (Table 2). Increasing SA concentration had a significant effect on FRAP activity of young ginger. The FRAP values for the leaf extracts of both varieties treated with SA were significantly lower than α-tocopherol (953 μmol Fe (II)/g), but higher than that of BHT (611.82 μmol Fe (II)/g). It was reported that the effect of antioxidant scavenging is due to hydrogen donating ability [28,29]. The FRAP assay has been used widely to estimate the antioxidant component/power of dietary polyphenols [30]. It is evident that foliar application of SA significantly enhanced the content of some flavonoids in both ginger varieties, and the high flavonoid content was associated with high antioxidant activity. In a previous study, a strong positive relationship between total flavonoid content and antioxidant activity was reported, which appears to be the trend in many plant species [31].

This study has shown that ginger has good free radical scavenging ability and therefore can be used as a radical inhibitor or scavenger, possibly acting as a primary antioxidant. Additionally, foliar application of SA can enhance the antioxidant activity of the ginger leaf extracts.

#### 2.3.2. DPPH Antioxidant Activity

**Figure 3:**
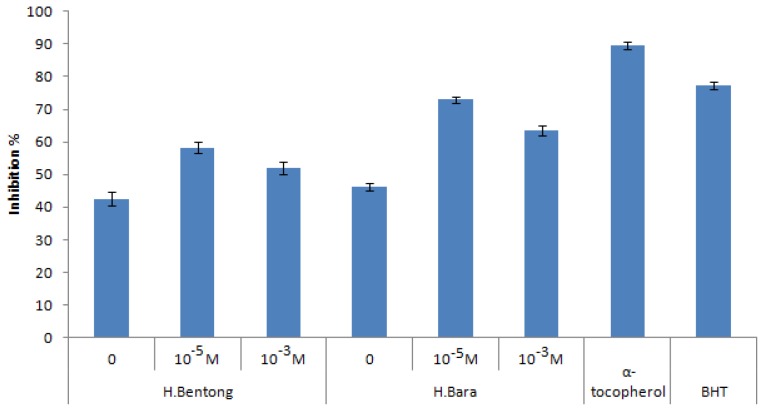
1,1-Diphenyl-2-picrylhydrazyl (DPPH) scavenging activities of two varieties of ginger treated with different concentration of SA compared with positive controls (α-tocopherol and BHT). Error bar represents standard error of means.

DPPH antioxidant activity was highest in the leaves of both varieties treated with SA compared to non treated plants (Figure 3). The effects on DPPH were contributed by SA treatments (*p* ≤ 0.05). At a concentration of 350 μg mL^−1^, the DPPH antioxidant activity recorded the highest value of 58.30%–72.90% with the 10^−5^ M SA treatment, followed by the 10^−3^ M SA (52.14%–63.66%) treatment, while the lowest value was obtained in the nontreated plants (42.50%–46.73%). However, DPPH radical scavenging ability of the plant extracts was lower than those of butylated hydroxyl toluene (BHT; 77.17%) and α-tocopherol (89.6%) registered at 350 μg mL^−1^. The highest value of DPPH activity was observed in H. Bara extracts (72.90%) when plants were treated with 10^−5^ M SA. This study showed that H. Bara extract had a good free radical scavenging activity, and hence can be used as a radical scavenger, acting possibly as a primary antioxidant. These results also imply that the 10^−5^ M SA treatment could significantly reduce the DPPH radical scavenging activity of the Malaysian ginger varieties. The principle involved is that in the presence of a molecule consisting of a stable free radical (DPPH), an antioxidant with the ability to donate a hydrogen atom will quench the stable free radical, a process which is associated with a change in absorption that can be translated spectrophotometrically.

**Figure 4:**
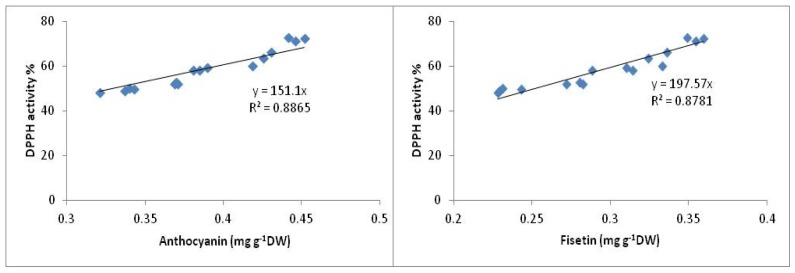
Relationship between anthocyanin and fisetin with DPPH activity in ginger varieties.

To date, more than 8000 phenolic compounds are known in plants, of which almost two-thirds belong to the predominantly water soluble flavonoid antioxidant family. Fisetin and anthocyanin were reported as potent antioxidant compounds with high antioxidant activity (DPPH activity) [32–34], and in the current study high concentrations of fisetin and anthocyanin were observed in ginger treated with 10^−5^ M SA. The high antioxidant activity in ginger varieties observed with the 10^−5^ M SA treatment is attributed to the high concentrations of anthocyanin and fisetin in these varieties. In this study, positive and significant (*p* < 0.05) correlations have been observed between anthocyanin (*R*^2^ = 0.88) and fisetin (*R*^2^ = 0.87) content with DPPH activity (Figure 4). The present findings are consistent with other research reports which found positive and significant correlations between anthocyanin and fisetin with antioxidant activity in medicinal plants [35,36]. This effect maybe due to the electron-donating ability of these compounds.

### 2.4. Anticancer Activity

The two ginger varieties were found to express MCF-7 and MDA-MB-231 cancer inhibitory activity when tested at concentrations of 4.6875–300 μg mL^−1^ (Table 3). Significant tumor inhibition was observed at 37.5 μg mL^−1^. At a concentration of 37.5 μg mL^−1^ most of the extracts exhibited strong anticancer activity towards MCF-7 and MDA-MB-231 cells. Maximum MCF-7 and MDA-MB-231 cell line inhibition was observed with H. Bara with values of 61.53% and 59.88%, respectively when treated with 10^−5^ M SA, while minimum MDA-MB-231 cell line inhibition was observed with the untreated H. Bentong (with values of 45.30% and 40.64%, respectively). MCF-7 and MDA-MB-231 cell lines treated with tamoxifen (positive control) showed 77.44% and 73.82% inhibition at the same concentration. Based on several *in vivo* and *in vitro* studies, many mechanisms of anticancer action may be involved. These include cell cycle arrest, carcinogen inactivation, antiproliferation, inhibition of angiogenesis, induction of apoptosis and differentiation, antioxidation and reversal of multi-drug resistance or a combination of these mechanisms [37–39]. Flavonoids are among the best candidates for mediating the protective effect of diets rich in fruits and vegetables with respect to colorectal cancer. Additional information on their effects on cancer cells and their mechanisms of action were obtained by adding, a series of related flavonoids to cultures of cancer cells; all flavonoid compounds were found to increase growth inhibition and cell loss at concentrations of 1 to 100 mM, with relative effectiveness being fisetin > kaempferol [40]. Hence, flavonoid compounds could probably be responsible for the anticancer activity of *Z. officinale*. According to the American National Cancer Institute, the criterion of normal cell viability for crude extracts of herbs is 76% [41]. This implies that extracts of herbs that show cell viability lower than this range are suitable for human consumption and are not harmful. Anthocyanin is a potent antioxidant [42–45]. Anthocyanin fractions extracted from different sources, including grape rinds and red rice [46], flower petals [47], Vaccinium species [48], red soyabeans and red beans [49], different cherry and berry extracts [50–52], and purple corn [53], have demonstrated anticancer activity. Katsube *et al.*[51] reported HCT-116 colon cancer cells were inhibited by anthocyanin-containing berry extracts including cowberry, blueberry, strawberry and bilberry extracts [51]. Similarly, tart cherry anthocyanins were shown to inhibit the growth of human colon cancer cell lines HCT116 and HT-29 [52]. Fisetin is found in several plants, fruits, vegetables, nuts and wine [54] and displays a variety of biological effects including anti-inflammatory, antioxidant [55,56], anti-carcinogenic and *in vitro* anti-angiogenesis [57]. *In vivo*, fisetin has recently been shown to possess interesting anticancer activity in mice bearing prostate tumours [58], lung carcinoma [59] and human embryonal carcinoma [60]. The *in vivo* mechanism of action appears rather complex and may include anti-angiogenic, anti-metastatic and anti-androgenic activities [61].

In the current study the highest values of both fisetin and athocyanin were detected in ginger varieties treated with 10^−5^ M SA. Meanwhile, the highest anticancer activity against MCF-7 and MDA-MB-231 cell lines has been observed with extracts of H. Bara treated with 10^−5^ M SA. This suggests that high anticancer activity in these varieties may be attributed to the high concentrations of potent anticancer components such as fisetin and anthocyanin. However, more research needs to be undertaken before the association between these flavonoids and anticancer activity in ginger varieties is more clearly understood. In varieties treated with SA the value of IC_50_ decreased significantly compared to non treated plants. The IC_50_ values of Halia Bara extract (not treated with SA) against MCF-7 and MDA-MB-231 cells were 39.1 and 42.1 μg mL^−1^, respectively (Table 4). In H. Bara treated with 10^−5^ M SA, the IC_50_ value decreased to 31.5 and 40.6 μg mL^−1^ for MCF-7 and MDA-MB-231 respectively; while the IC_50_ values of tamoxifen as a positive control for MCF-7 and MDA-MB-231 cells were 17.4 and 19.5 μg mL^−1^, respectively.

## 3. Experimental Section

### 3.1. Plant Material and Maintenance

The rhizomes of ginger varieties Halia Bentong and Halia Bara (*Zingiber officinale*) were collected from the ginger planting area in Bentong Village, Malaysia. Voucher specimens were identified by the herbarium of the University Putra Malaysia, Selangor, Malaysia (H. Bentong Stone 6030 (KLU) and H. Bara Stone 7233 (KLU)). Rhizomes were sprouted for two weeks in 10 cm diameter pots filled with peat. They were then transferred to polyethylene bags filled with a soilless mixture consisting of burnt rice husk and coco peat (1:1). The plants were grown in a glasshouse at the Universiti Putra Malaysia (UPM) glasshouse complex. The seedlings were raised in specially constructed growth chambers receiving 12-h photoperiod and average photosynthetic photon flux density of 310 μmol m^−2^ s^−1^. Day and night temperatures were maintained at 30 ± 1.0 °C and 20 ± 1.5 °C, respectively, and relative humidity of between 70% and 80%. At the second leaf stage, the ginger seedlings were sprayed with two concentrations (10^−3^ or 10^−5^ M) of salicylic acid solution (SA; 2-hydroxybenzoic acid +100 μL dimethyl sulfoxide +0.02% polyoxyethylenesorbitan monolaurate, Tween 20, Sigma Chemicals; pH 6.5). Control plants were sprayed with the same solution but without SA. Plants were sprayed early in the morning with 10 mL of the respective solutions once a week for four weeks.

### 3.2. Extract Preparation

Leaf samples (0.25 g) were extracted with 20 mL of methanol, and 5 mL of 6 M HCl was added to each extract to give a total volume of 25 mL. The extracts were then refluxed at 90 °C for 2 h. Aliquots of 500 μL were taken before and after hydrolysis, filtered through a 0.45 μm filter, and analyzed for flavonoids using High Performance Liquid Chromatography (HPLC) [62].

### 3.3. High Performance Liquid Chromatography (HPLC) Analysis

#### 3.3.1. Standard Curve Preparation

The standard stock solutions were prepared by dissolving standards in methanol to 100 μg mL^−1^. For the calibration curves, four additional concentrations (20, 40, 60 and 80 μg mL^−1^) were prepared by the dilution of the stock solutions with methanol.

#### 3.3.2. Chromatography Conditions

Reversed-phase HPLC was used to assay flavonoid composition. The Agilent HPLC system used consisted of a Model 1100 pump equipped with a multi-solvent delivery system, an L-7400 ultraviolet (UV) detector, and fitted with an Agilent C18 (5 μm, 4.6 mm internal diameter 250 mm) column. The mobile phase consisted of: (A) 2% acetic acid (CH_3_COOH) and (B) 0.5% acetic acid-acetonitrile (CH_3_CN), (50:50 *v*/*v*). The mobile phase was filtered under vacuum through a 0.45 um membrane filter before use. Gradient elution was performed as follows: 0 min, 95:5; 10 min, 90:10; 40 min, 60:40, 55 min, 45:55; 60 min, 20:80; and 65 min, 0:100. The flow rate was maintained at 1 mL min^−1^ and UV absorbance was measured at 280–365 nm. The operating temperature was maintained at room temperature [63]. Identification of the flavonoids was achieved by comparison of retention times with standards, UV spectra and UV absorbance ratios after co-injection of samples and standards. The standards were purchased from Sigma–Aldrich (St. Louis, MO, USA).

### 3.4. Chalcone Synthase (CHS) Assay

CHS activity was assayed spectrophotometrically as described in Obinata *et al.*[17]. Enzymes were extracted at 4 °C by homogenizing the frozen harvested cells (0.4 g) in 1 mL of 0.1 M borate buffer (pH 8.8) containing 1 mM 2-mercaptoethanol with a homogenizer (Polytron). The homogenates were treated with 0.1 g of Dowex l × 4 for 10 min and the cell debris and resin were removed by centrifugation at 15,000 rpm for 10 min. A 0.2 g sample of Dowex l × 4 resin was added to the supernatant and treated for another 20 min. The resin was then removed by centrifugation at 15,000 rpm for 15 min. The resultant supernatant was used in the CHS assay. The CHS assay was performed with 100 μL of enzyme extract mixed with 1.89 mL of 50 mM Tris-HCI buffer, pH 7.6, containing 10 mM KCN. The enzyme reaction was allowed to proceed for 1 min at 30 °C after adding 10 mg chalcone to 10 μL ethylene glycol monomethylether. The activity was determined by measuring the absorbance at 370 nm.

### 3.5. Determination of Antioxidant Activities

#### 3.5.1. Ferric Reducing Antioxidant Potential (FRAP) Assay

The stock solutions consisted of 300 mM acetate buffer, 10 mM TPTZ (2,4,6-tripyridyl-*S*-triazine) solution in 40 mM HCl, and 20 mM FeCl_3_ solution. Acetate buffer (25 mL) and TPTZ (2.5 mL) were mixed, and 2.5 mL FeCl_3_ added. Leaf extract (150 μL) was added to 2850 μL of the FRAP solution and kept for 30 min in the dark place. The absorbance of solution was measured at 593 nm using a spectrophotometer (U-2001, Hitachi Instruments Inc., Tokyo, Japan) [64].

#### 3.5.2. 1,1-Diphenyl-2-picrylhydrazyl (DPPH) Assay

1,1-Diphenyl-2-picrylhydrazyl (DPPH) was purchased from Sigma–Aldrich. Butylated hydroxytoluene (BHT) and α-tocopherol were purchased from Merck (India). The radical scavenging ability was determined using the method described in Mensor *et al.*[65]. Briefly, an alcohol solution of DPPH (1 mL, 3 mg mL^−1^) was added to 2.5 mL samples containing different concentrations of extracts originating from different parts of the ginger varieties. The samples were first kept in the dark at room temperature and their absorbance was read at 518 nm after 30 min. The antiradical activity was determined using the following formula:

(1) Percent (%) inhibition of DPPH activity=[(A0-A1)/A0]×100%

Where *A*0 is the absorbance value of the blank sample or control reaction, and *A*1 is the absorbance value of the test sample. The optic density of the samples and controls were measured in comparison to ethanol. BHT (butylhydroxytoluene) and α-tocopherol, were used as positive controls.

### 3.6. Determination of Anticancer Activity

#### 3.6.1. Cell Culture and Treatment

Human breast carcinoma cell lines (MCF-7 and MDA-MB-231) were cultured in 100 μL of RPMI 1640 media (Roswell Park Memorial Institute) containing 10% fetal bovine serum (FBS). MCF-7 and MDA-MB-231 cells were incubated overnight at 37 °C in 5% CO_2_ for cell attachment.

#### 3.6.2. MTT (3-(4,5-Dimethylthiazol-2-yl)-2,5-diphenyltetrazolium bromide) Assay

The assay was conducted as follows: Cancer cells were seeded in 96-well plates at a density of 1 × 104 cells/well in 100 μL RPMI. At 24 h after seeding, the medium was removed and the cells were incubated for 3 days with RPMI in the absence or presence of various concentrations of ginger extracts. Ginger extract concentrations used ranged from 4.6875, 9.375, 18.75, 37.5, 75, 150 to 300 μg mL^−1^. After incubation, 20 μL of MTT [3-(4,5-dimethylthiazol-2-yl)-2,5-diphenyltetrazolium bromide] reagent was added into each well. The plate was incubated again for 4 h in a CO_2_ incubator at 37 °C. The resulting MTT–products were determined by measuring the absorbance at 570 nm using ELISA reader [66]. Each point represents the mean of triplicate experiments. The cell viability was determined using the formula:

(2) Viability (%)=(optical density of sample/optical density of control)×100

### 3.7. Statistical Analysis

Data were analyzed using the Statistical Analysis System (SAS, system 9.0; SAS Institute, Inc.: Cary, NC, USA, 2002). Means separation was performed using the Duncan multiple range test. The experimental results were expressed as mean ± standard deviation of three replicates. Mean differences at *p* ≤ 0.05 were regarded as significant.

## 4. Conclusions

This study demonstrated that foliar application of SA could be promoting the production of secondary metabolites and as a consequence, the antioxidant and anticancer properties of ginger varieties have been improved. SA is one of the special elicitors in the study of elicitation of secondary compounds through physiological pathways. Treatment of H. Bentong and H. Bara with SA improved production of fisetin and anthocyanin who have potent antioxidant activity and which were not detected in control plants. SA can act not only as an inducer but also as an inhibitor of secondary compound production [67] and in this study reduction of rutin and apigenin in both varieties was observed. In agreement with Nicholson and Hammerschmidt [68], an increase in the activity of CHS can be considered as a biochemical marker for resistance, given that this enzyme is the key for the necessary synthesis of flavonoid compounds. It would appear that foliar application of SA not only significantly enhances biomass production in ginger varieties, but that it also slightly increases the concentrations of several therapeutic compounds. Halia Bentong and Halia Bara exhibited promising anticancer activity on human breast cancer cell lines (MCF-7 and MDA-MB-231). The leaf extracts of these varieties contained appreciable amounts of effective flavonoid compounds like fisetin, morien, myreciten and anthocyanin, which is a potent agent for breast cancer growth inhibition. Subsequently, our MTT assay indicated that enriched Halia Bara leaf with 10^−5^ M of SA is a potential source of anticancer therapeutic compounds. It seems that SA could be used to enhance phyrochemical production and the pharmaceutical quality of ginger.

## Figures and Tables

**Table 1:** High performance liquid chromatography analysis of ginger (*Zingiber officinale*) varieties treated with salicylic acid (SA).

Compounds	Halia Bentong			Halia Bara		
	
	Control	SA 10^−5^	SA 10^−3^	Control	SA 10^−5^	SA 10^−3^
Rutin	0.893 ± 0.03 ^b,c^	0.736 ± 0.09 ^c^	0.79 ± 0.06 ^c^	1.13 ± 0.12 ^a^	0.883 ± 0.07 ^b,c^	0.993 ± 0.07 ^a,b^
Apigenin	0.384 ± 0.049 ^c^	0.276 ± 0.08 ^d^	0.305 ± 0.09 ^d^	0.553 ± 0.06 ^a^	0.45 ± 0.06 ^b^	0.55 ± 0.04 ^a^
Myricetin	0.04 ± 0.009 ^b^	0.088 ± 0.009 ^a,b^	0.059 ± 0.018 ^b^	0.06 ± 0.001 ^b^	0.112 ± 0.004 ^a^	0.074 ± 0.006 ^a,b^
Naringenin	0.227 ± 0.049 ^a^	0.29 ± 0.1 ^a^	0.259 ± 0.045 ^a^	0.259 ± 0.033 ^a^	0.303 ± 0.097 ^a^	0.304 ± 0.02 ^a^
Fisetin	ND	0.237 ± 0.017 ^c^	0.228 ± 0.03 ^c^	ND	0.359 ± 0.046 ^a^	0.304 ± 0.01 ^b^
Morien	0.117 ± 0.02 ^a,b^	0.173 ± 0.055 ^a,b^	0.158 ± 0.042 ^a,b^	0.102 ± 0.042 ^b^	0.193 ± 0.03 ^a^	0.182 ± 0.017 ^a^
Anthocyanin	ND	0.381 ± 0.05 ^b^	0.369 ± 0.053 ^b^	ND	0.442 ± 0.041 ^a^	0.426 ± 0.122 ^a^

All analyses are the mean of triplicate measurements ±standard deviation; Results expressed in mg g^−1^ DW; Means not sharing a common single letter were significantly different at *p* ≤ 0.05.; ND: not detected.

**Table 2:** Antioxidant activity (FRAP) in two varieties of *Zingiber officinale* treated with different concentration of salicylic acid (SA).

Variety	SA (M)	FRAP (μmol Fe (II)/g)
H. Bentong	0	476.22 + 10.19 ^e^
	10^−5^	644.7 + 32.7 ^c^
	10^−3^	521.3 + 27.1 ^d^
	0	509.4 + 14.26 ^d^
H. Bara	10^−5^	793.26 + 33.2 ^a^
	10^−3^	716.62 + 32.5 ^b^
Positive controls	BHT	611.82 + 15.2
	α-tocopherol	887.34 + 29.5

All analyses are the mean of triplicate measurements ± standard deviation. Means not sharing a common letter were significantly different at *p* ≤ 0.05.

**Table 3:** Anticancer activities (cell viability) and percent of inhibition of ginger extracts towards MCF-7 and MDA-MB-231 cell lines as determined by the MTT assay (at concentration 37.5 μg mL^−1^).

Variety	SA (M)	MCF-7	MDA-MB-231	Inhibition % (MCF-7)	Inhibition % (MDA-MB-231)
H. Bentong	0	54.7 ± 2.17 ^a^	59.36 ± 2.2 ^a^	45.3	40.64
	10^−5^	41.08 ± 1.56 ^b^	45.3 ± 1.63 ^b^	58.92	54.7
	10^−3^	47.44 ± 1.64 ^b^	48.69 ± 1.77 ^b^	52.56	51.31
H. Bara	0	51.63 ± 1.92 ^a^	56.2 ± 2.11 ^a^	48.37	43.8
	10^−5^	38.47 ± 1.15 ^c^	40.12 ± 1.4 ^c^	61.53	59.88
	10^−3^	42.28 ± 1.68 ^b^	47.18 ± 1.58 ^c^	58.72	52.82

Positive control	Tamoxifen	22.56 ± 1.07	26.18 ± 1.27	77.44	73.82

All analyses are the mean of triplicate measurements ± standard deviation. Results expressed in percent of cell viability. Means not sharing a common letter were significantly different at *p* ≤ 0.05.

**Table 4:** IC_50_ values of ginger extracts towards MCF-7 and MDA-MB-231 cancer cell lines as determined by the MTT assay.

Variety	SA (M)	MCF-7	MDA-MB-231
H. Bentong	0	50.6 ± 1.45 ^a^	54.7 ± 1.74 ^a^
	10^−5^	42.5 ± 1.33 ^b^	48.8 ± 1.3b ^c^
	10^−3^	44.4 ± 1.72 ^b^	50.6 ± 1.28 ^b^
H. Bara	0	39.1 ± 1.18 ^c^	45.2 ± 1.14 ^d^
	10^−5^	31.5 ± 1.66 ^d^	40.6 ± 1.06 ^f^
	10^−3^	33.6 ± 1.2 ^d^	44.5 ± 1.66 ^e^

Tamoxifen		17.4 ± 2.16	19.5 ± 1.88

All analyses are the mean of triplicate measurements ± standard deviation. Results expressed in percent of cell viability. Means not sharing a common letter were significantly different at *p* ≤ 0.05.
